# Delinquents

**Published:** 1872-10

**Authors:** 


### DELINQUENTS.

We are authorized to state that many of our subscribers have, so far, failed to remit their dues to the COMPANION. On this account, and the disadvantages growing out of it, the publisher has already advanced the full amount of the subscription price of each subscriber—the year having nearly expired. The subscription price leaves scarcely a margin for publishing—hence, it is important to the publisher, that *all* should pay. We have no reason to doubt but that all those who have failed to pay will do so now, as accounts were forwarded them in the last number, and they understand the publisher's wants fully.

We urge our friends, who have not paid, to forward their dues at once. Do not neglect this—as we feel it is neglect rather than a desire on their part in not attending to this small (but important) matter. Come friends, send in your money and cancel a debt you ought *now* to pay.

